# Immunosuppression for adult steroid-dependent or frequently relapsing nephrotic syndrome: A systematic review and meta-analysis

**DOI:** 10.1371/journal.pone.0307981

**Published:** 2024-07-31

**Authors:** Zhi Yong Wong, Chiu Yee Teo, Yan Qi Fiona Wong, Ka Ting Ng, Soo Kun Lim

**Affiliations:** 1 Faculty of Medicine, University of Malaya, Kuala Lumpur, Malaysia; 2 Faculty of Medicine, Department of Anesthesiology, University of Malaya, Kuala Lumpur, Malaysia; 3 Faculty of Medicine, Department of Medicine, University of Malaya, Kuala Lumpur, Malaysia; Istituto Di Ricerche Farmacologiche Mario Negri, ITALY

## Abstract

**Objective:**

There is limited evidence on which immunosuppressive agents produce the best outcomes for adult patients with steroid-dependent or frequently relapsing nephrotic syndrome (SDNS/FRNS). This review compares the remission rate and adverse effects of various immunosuppressants used.

**Methods:**

Studies of adult patients with biopsy-proven SDNS/FRNS, administered any immunosuppressive agents and reported complete remission results as one of the clinical outcomes were included. Articles were independently screened by two researchers. ROBINS-I was used for risk of bias assessment. Random-effects model was used for statistical analysis and corresponding 95% confidence intervals (CIs) were calculated.

**Results:**

574 patients across 28 studies were included in the analysis. Patients receiving rituximab have a complete remission rate of 89% (95% CI = 83% to 94%; τ2 = 0.0070; I2 = 62%; overall p < 0.01, low certainty) and adverse event rate of 0.26, cyclosporine (CR 40%; 95% CI = 21% to 59%; τ2 = 0.0205; I2 = 55%; overall p = 0.08, low certainty), tacrolimus (CR 84%; 95% CI = 70% to 98%; τ2 = 0.0060; I2 = 33%; overall p = 0.21, moderate certainty), mycophenolate mofetil (CR 82%; 95% CI = 74% to 90%; τ2 < 0.0001; I2 = 15%; overall p = 0.32, moderate certainty) and cyclophosphamide (CR 79%; 95% CI = 69% to 89%; τ2 = 0; I2 = 0%; overall p = 0.52, moderate certainty).

**Conclusion:**

Among the commonly used immunosuppressive agents, only rituximab has a statistically significant effect in achieving complete remission among patients with SDNS/FRNS and has a relatively good safety profile, but this is limited by low quality of evidence with high degree of heterogeneity causing a lack of statistical power.

## Introduction

Since the first use of corticosteroids to treat childhood nephrotic syndrome in 1950 [1], glucocorticoids have been widely regarded as an initial therapy for adult patients with nephrotic syndrome over the years [2–8]. In pediatric populations, steroid-dependent patients who relapse during therapy or within two weeks of discontinuation often experience severe side effects from long-term corticosteroid use, such as development of cushingoid features, diabetes mellitus, hypertension, osteoporosis, hematological abnormalities such as leukocytosis or neutrophilia and behavioral disturbances [9]. Achieving a remission rate of 75%–95% among adults with minimal change disease (MCD) [10] and 40–60% among adults with focal segmental glomerulosclerosis (FSGS) [11], steroid dependent adults often experience severe side effects as well. This has subsequently led to an increasing interest towards corticosteroid-sparing agents as alternatives to treatment for avoidance of steroid toxicity.

Identified in the 1960s, cyclophosphamide is among the earliest immunosuppressants [12] known to reduce proteinuria and number of relapses among patients with nephrotic syndrome [13] that is still currently used to date. In 1986, cyclosporine A (CsA) was reported to be first used among adults with difficult to treat nephrotic syndrome [14]. It was until the early 1990s before tacrolimus was used for adult nephrotic syndrome [15–17] followed by the introduction of mycophenolate mofetil in 2000 [18]. The next was rituximab, first reported in 2007 [19] when administered in five patients with nephrotic syndrome.

With the introduction of immunosuppressants, multiple studies evaluating the efficacy of one agent over the other were established. The use of rituximab for steroid-dependent nephrotic syndrome is increasing, while alternatives such as cyclophosphamide, calcineurin inhibitors, and mycophenolate mofetil have also been recommended as the preferred medications in different clinical trials [20]. For patients with SDNS/FRNS, a network meta-analysis for children suggests that cyclophosphamide may be preferred initially while chlorambucil and rituximab may be acceptable medications [21]. Among adult patients with SDNS/FRNS, there has not been a meta-analysis comparing different immunosuppressant agents. This study aims to evaluate the effectiveness and safety of various immunosuppressants for adult patients with SDNS/FRNS to reduce steroid toxicity associated with conventional corticosteroid treatment.

## Materials and methods

This systematic review is registered in PROSPERO (CRD42023399790) and conducted in accordance with the Preferred Reporting Items for Systematic Reviews and Meta-Analyses (PRISMA) extension statement for reporting network meta-analyses 2020 [22] in S1 Fig.

### Search strategy

From the electronic databases Medline via PubMed, Embase, Cochrane Central Register of Controlled Trials (CENTRAL) and Web of Science, we looked for articles published from inception till 1 March 2024.

The main keywords used for the search include “nephrotic syndrome” OR “minimal change disease” OR “focal segmental glomerulosclerosis” to represent the patient population added with “immunosuppression therapy” OR its examples such as rituximab, tacrolimus, Mabthera, cyclophosphamide, cyclosporine, mycophenolic acid, mycophenolate added up with “steroid-dependent” OR “steroid-sensitive” OR “frequently relapsing”.

Truncations and subject headings or keywords were included in the search. Complete search strategies with details for each database containing when each source was last searched is available in a separate table in S1 Table.

### Eligibility criteria

Randomized controlled trials or observational studies that report data from adult patients aged 15 and above with biopsy-proven nephrotic syndrome, is steroid-dependent or frequently relapsing, administered any immunosuppressive agents in any dosage and reports complete remission results as one of the clinical outcomes were included.

Nephrotic syndrome in adults is defined, according to the Kidney Disease: Improving Global Outcomes (KDIGO), as patients with nephrotic range proteinuria characterized by >3.5g per 24 hours or urine protein-creatinine ratio of ≥ 300mg/mmol in the presence of hypoalbuminemia, oedema or hyperlipidemia [23]. Steroid dependence is defined as two consecutive relapses during therapy with prednisone/prednisolone either at full dose or during tapering or within 14 days of prednisone/prednisolone discontinuation [23]. Frequently relapsing is defined as steroid-sensitive nephrotic syndrome (SSNS) with 2 or more relapses within 6 months, or 4 or more relapses within a 12-month period.

We excluded patients with steroid resistant nephrotic syndrome, concomitant infections, post-transplant patients and concurrent comorbid conditions or systemic diseases that could have a pathogenic relationship with the nephrotic syndrome, case reports, review articles, letters to editors and articles not in English.

### Study selection and data extraction

Duplicates were automatically removed using EndNote version 21.1 (Clarivate, Philadelphia, PA), and the remaining results were manually screened for duplicates. Two reviewers (Z.Y.W. and C.Y.T.) independently screened study titles and abstracts to exclude ineligible studies. The full texts of selected studies from the first screening were then retrieved and assessed. Any discrepancies were resolved after discussion with another two authors (K.T.N. and S.K.L). Two researchers (Z.Y.W. and C.Y.T) extracted data from the included studies according to a predetermined proforma in Microsoft Excel. Data on study characteristics consisted of study design, country, funding, and inclusion/exclusion criteria. Data on participants’ characteristics consisted of sample size, mean age, mean follow-up duration, baseline serum creatinine level and previous therapies. Data on study results include percentage of complete remission, partial remission, duration of remission and rate of relapse.

### Risk of bias assessment

Risk of Bias in Nonrandomized Studies of Interventions (ROBINS-I) tool [24] was used for nonrandomized included studies where two reviewers (Z.Y.W. and C.Y.T.) independently judged all seven domains as having low, moderate, serious, or critical risk of bias, or no information. In the event of discrepancies, they were resolved by the independent opinion of a third reviewer (K.T.N. or S.K.L.). A study would be judged as having an overall low risk of bias if all the domains were judged as low risk. A study would be considered as having critical risk of bias if at least one domain was judged as high risk of bias. Certainty of evidence was assessed using the Grading of Recommendations Assessment, Development and Evaluation [25] and rated as very low, low, moderate or high based on the assessment of the domains for indirectness, publication bias, risk of bias, incoherence, intransitivity, imprecision and inconsistency.

### Outcomes of interest

The primary outcome of interest is rate of complete remission at the time of last follow up, defined as a proteinuria value ≤ 300 mg per day. Secondary outcomes include rate of partial remission, defined as a decrease of the initial urinary protein loss by at least 50% and a proteinuria value ≤ 3.5 g per day and adverse events.

### Data analysis

Statistical analysis of the pooled outcomes of interests was performed using R Statistics software. The random-effects model was used and corresponding 95% confidence intervals (CIs) was calculated for outcomes. Statistical heterogeneity of the results in the included studies was assessed by I2 statistic. Heterogeneity is significant when the I2 statistic is ≥ 50%. Assessment for publication bias was qualitative through visual inspection for funnel plot asymmetry in S2 Fig. An overall p value of < 0.05 is considered statistically significant.

## Results

### Search results

**Fig 1:**
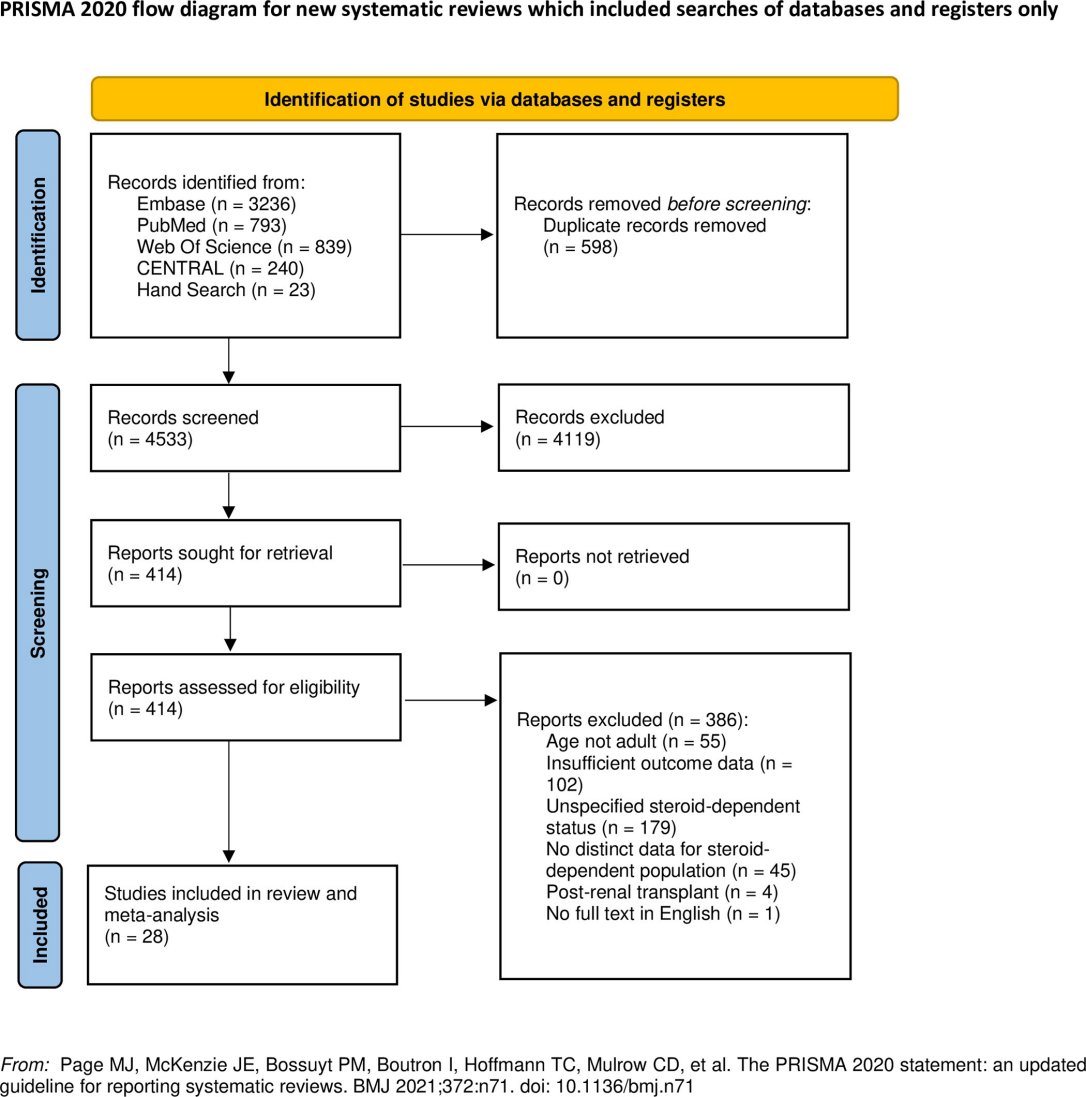
PRISMA 2020 flowchart.

The literature searches retrieved a total of 5,131 references, which reduced to 4,533 after removal of duplicates. The titles and abstracts were screened, and we subsequently removed 4,119 references. We assessed 414 full texts for eligibility, of which we excluded 386 references. A total of 28 studies [26–53] were included in this systematic review and meta-analysis with full details available in a separate table in S2 Table. A flowchart of the number of articles included or excluded at each stage is available in Fig 1 [22].

### Study and patient characteristics

Our study included a total of 574 patients across 28 studies [26–53], of which 330 from 19 studies [27–31,33–34,36–40,42–44,46–47,49,52] received rituximab, 61 from four studies [32,41,51,53] received cyclosporine, 59 received from five studies [26,33,35,48,51] received cyclophosphamide, 90 from four studies [26,33,50,51] received mycophenolate mofetil, and 34 from four studies [26,45,48,51] received tacrolimus. There are 9 prospective studies [27,39,42,44–46,48–49,52] and 19 retrospective studies [26,28–38, 40–41,43,47,50–51,53]. Five studies were conducted in USA [28,33,36–37,51], six studies in India [26,31,35,45,49,52], five in China [30,41,44,47,48], four studies in Japan [34,39,42,46], two each in France [40,43] and Spain [29,50] as well as one each in Netherlands [38], Sweden [27], Tunisia [32] and Pakistan [53].

21 studies [28–35,37,39–40,42–48,50–51,53] reported the baseline creatinine level of patients. 9 studies [28,29,31,32,35,40,43,51,53] reported a mean baseline creatinine of 1.08 mg/dL, 2 [33,34] studies reported a median baseline creatinine level of 0.69mg/dL and 0.9 mg/dL respectively whereas the other 10 studies [30,37,39,42,44–48,50] did not mention whether baseline creatinine level was calculated in mean or median. Extracted data from 11 studies [26–28,35,41,46–48,50–52] reported a mean rate of relapse of 33.33%, while the duration of remission from 6 studies [27,37,41,44,48,51] ranges between 1 to 43 months.

For previous therapies, 8 studies [27,28,34,37,38,43,48,51] reported the use of cyclophosphamide, 11 studies [27,28,31,34,37–39,43,48,51,53] reported the use of cyclosporine, 10 studies [27,28,34,37–40,43,47,48] reported the use of mycophenolate mofetil, 7 studies [27,28,31,34,37,39,43] reported the use of tacrolimus, 3 studies [27,38,43] reported the use of levamisole and 2 studies [34,39] reported the use of mizoribine. Azathioprine, abatacept, rituximab, mechlorethamine, basiliximab, chimeric antigen receptor (CAR), chlorambucil, pefloxacin were reported in 1 study respectively [28,30,43].

### Risk of bias assessment

Table 1 shows the results from the risk of bias assessment. All 28 studies were assessed to be of medium risk of bias. No studies were considered at severe or critical risk of bias. The risk of bias was mainly related to confounding effects and deviation from intended interventions.

**Table 1:** Risk of bias assessment.

Study	Domain 1: Risk of bias due to confounding	Domain 2: Bias in selection of participants into the study	Domain 3: Bias in classification of interventions	Domain 4: Bias due to deviations from intended interventions	Domain 5: Bias due to missing data	Domain 6: Bias in measurement of outcomes	Domain 7: Bias in selection of the reported result	Overall judgement
Aarthi et al., 2019 [26]	Moderate	Low	Moderate	Moderate	Low	Low	Low	Moderate
Bruchfeld et al., 2014 [27]	Moderate	Low	Low	Moderate	Low	Low	Low	Moderate
Cortazar et al., 2018 [28]	Moderate	Low	Low	Moderate	Low	Low	Low	Moderate
DaSilva et al., 2017 [29]	Moderate	Low	Low	Moderate	Low	Low	Low	Moderate
Girimaji et al., 2021 [31]	Moderate	Low	Low	Moderate	Low	Low	Low	Moderate
Gorsane et al., 2016 [32]	Moderate	Low	Low	Moderate	Moderate	Low	Low	Moderate
Heybeli et al., 2021 [33]	Moderate	Low	Moderate	Moderate	Low	Low	Low	Moderate
Katsuno et al., 2019 [34]	Moderate	Low	Low	Moderate	Low	Low	Low	Moderate
Keskar et al., 2013 [35]	Moderate	Low	Moderate	Moderate	Low	Low	Low	Moderate
Kidd & Bean, 2018 [36]	Moderate	Low	Low	Moderate	Low	Low	Low	Moderate
Brown et al., 2017 [37]	Moderate	Low	Low	Moderate	Low	Low	Low	Moderate
Dekkers et al., 2015 [38]	Moderate	Low	Low	Moderate	Low	Low	Low	Moderate
Guitard et al., 2014 [40]	Moderate	Low	Low	Moderate	Low	Low	Low	Moderate
Miyabe et al., 2016 [42]	Moderate	Low	Low	Low	Low	Low	Low	Moderate
Munyentwali et al., 2013 [43]	Moderate	Low	Low	Low	Low	Low	Low	Moderate
Ren et al., 2017 [44]	Moderate	Low	Low	Low	Low	Low	Low	Moderate
Takei et al., 2013 [46]	Low	Moderate	Low	Moderate	Low	Low	Low	Moderate
Li et al., 2021 [47]	Moderate	Low	Moderate	Moderate	Low	Low	Low	Moderate
Li et al., 2008 [48]	Moderate	Low	Moderate	Moderate	Low	Low	Low	Moderate
Ramachandran et al., 2020 [52]	Moderate	Low	Low	Low	Low	Low	Low	Moderate
Ramachandran et al., 2019 [49]	Moderate	Low	Low	Low	Low	Low	Low	Moderate
Ramachandran et al., 2015 [45]	Moderate	Low	Low	Low	Low	Low	Low	Moderate
Sandoval et al., 2017 [50]	Moderate	Low	Low	Moderate	Low	Low	Low	Moderate
Waldman et al., 2007 [51]	Moderate	Low	Low	Moderate	Low	Low	Low	Moderate
Zhao et al., 2022 [41]	Moderate	Low	Moderate	Low	Low	Low	Low	Moderate
Iwabuchi et al., 2014 [39]	Moderate	Low	Low	Low	Low	Low	Low	Moderate
Jafry et al., 2023 [53]	Moderate	Low	Low	Low	Low	Low	Low	Moderate
Zhang et al., 2023 [30]	Moderate	Low	Low	Low	Low	Low	Low	Moderate

### Rate of complete remission

**Fig 2:**
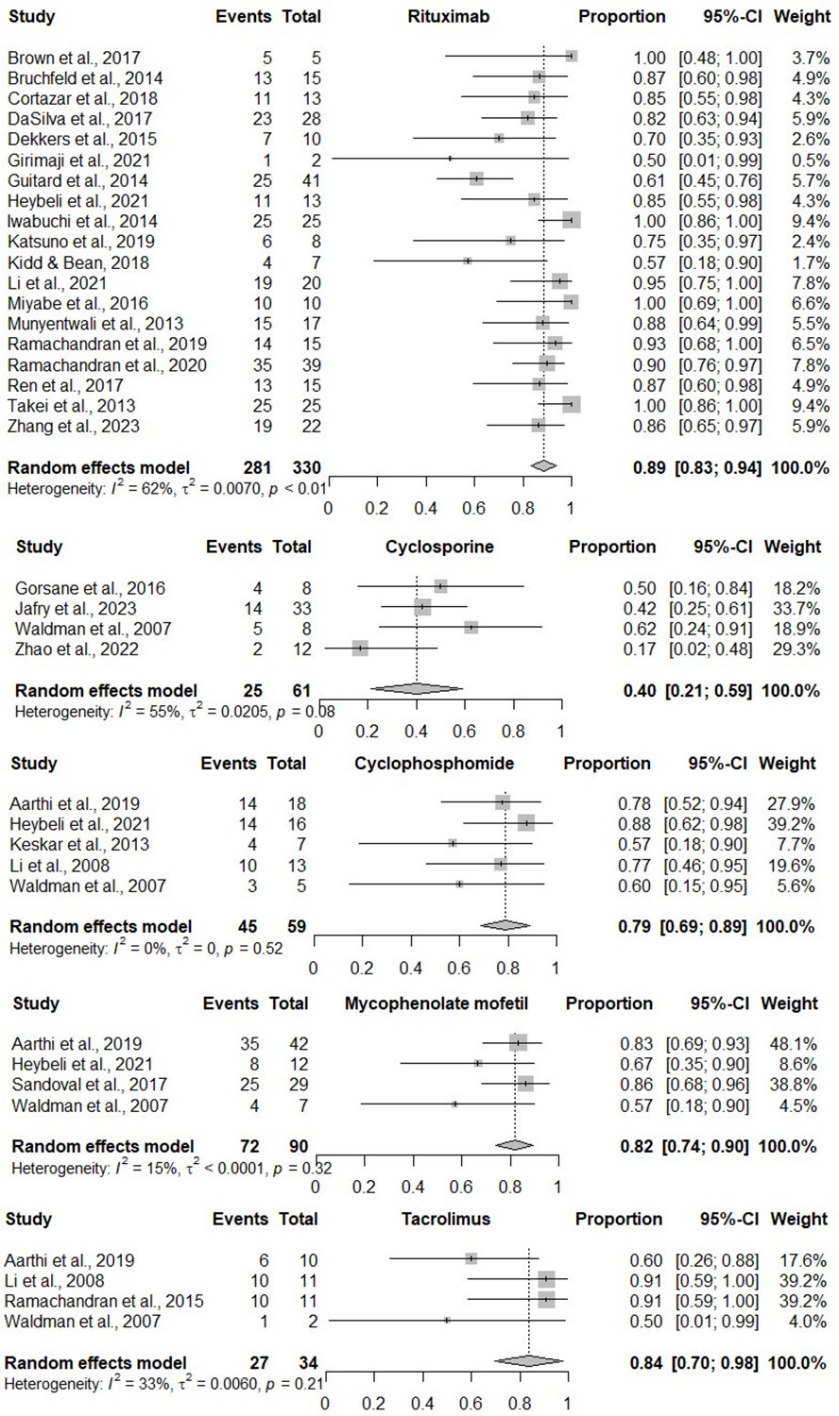
Pooled rate of complete remission according to immunosuppressive treatment.

All included studies [26–53] reported the rate of completed remission, which was pooled according to treatment options in Fig 2. According to the pooled results from 19 studies [27–31,33,34,36–40,42–44,46,47,49,52], patients receiving rituximab had a complete remission rate of 89% (95% CI = 83% to 94%; τ2 = 0.0070; I2 = 62%; overall p < 0.01, low certainty). Patients receiving cyclosporine had a complete remission rate of 40% (95% CI = 21% to 59%; τ2 = 0.0205; I2 = 55%; overall p = 0.08, low certainty) based on the results from four studies [32,41,51,53]. The heterogeneity of both pooled results from cyclosporine and rituximab studies are high I2 > 50%. Five studies [26,33,35,48,51] reported outcomes from using cyclophosphamide with a complete remission rate of 79% (95% CI = 69% to 89%; τ2 = 0; I2 = 0%; overall p = 0.52, moderate certainty). Four studies [26,33,50,51] reported outcomes from using mycophenolate mofetil with a complete remission rate of 82% (95% CI = 74% to 90%; τ2 < 0.0001; I2 = 15%; overall p = 0.32, moderate certainty). Four studies [26,45,48,51] reported outcomes from using tacrolimus with a complete remission rate of 84% (95% CI = 70% to 98%; τ2 = 0.0060; I2 = 33%; overall p = 0.21, moderate certainty). As the meta-analysis of cyclosporine, cyclophosphamide, tacrolimus and mycophenolate mofetil have less than 10 studies, the power of the test with funnel plot is too low to distinguish chance from real asymmetry [54]. Visual inspection of the funnel plot for rituximab revealed asymmetry [S2 Fig. Funnel Plot], which may be attributed to publication bias or heterogeneity arising from the differing tapering steroid dosages that could lead to smaller estimates of the effect of intervention.

### Rate of partial remission

**Fig 3:**
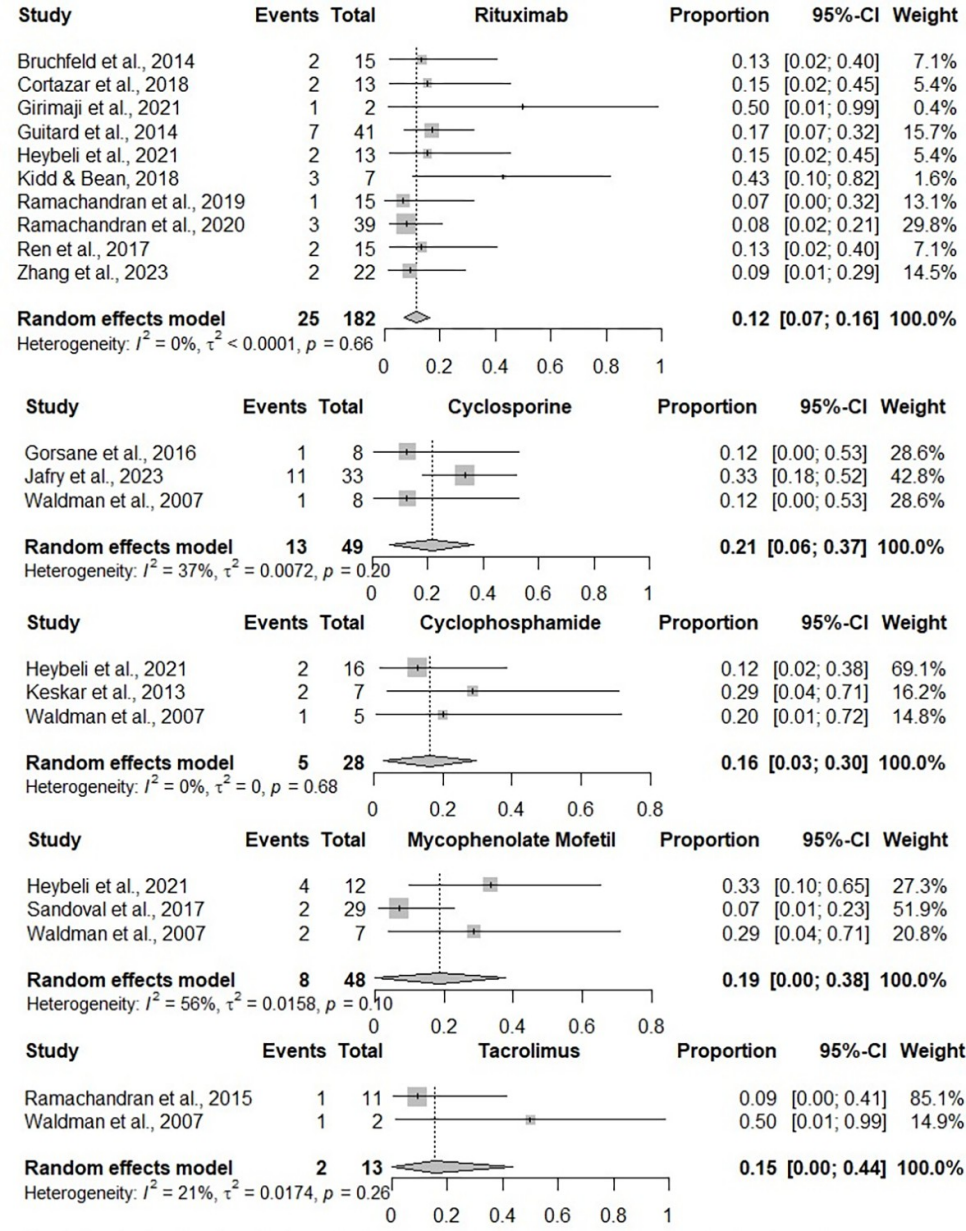
Pooled rate of partial remission according to immunosuppressive treatment.

16 studies [27,28,30–33,35,36,40,44,45,49–53] reported the rate of partial remission, which was pooled according to treatment options in Fig 3. Based on the pooled results from 10 studies [27,28,30,31,33,36,40,44,49,52], patients receiving rituximab had a partial remission rate of 12% (95% CI = 7% to 16%; τ2 < 0.0001; I2 = 0%; overall p = 0.66, low certainty). According to three studies [32,51,53], patients receiving cyclosporine had a partial remission rate of 21% (95% CI = 6% to 37%; τ2 = 0.0072; I2 = 37%; overall p = 0.20, moderate certainty). Three studies [33,35,51] reported outcomes from using cyclophosphamide with a partial remission rate of 16% (95% CI = 3% to 30%; τ2 = 0; I2 = 0%; overall p = 0.68, moderate certainty). Three studies [33,50,51] reported outcomes from using mycophenolate mofetil with a partial remission rate of 19% (95% CI = 0% to 38%; τ2 = 0.0158; I2 = 56%; overall p = 0.10, low certainty). Two studies [45,51] reported outcomes from using tacrolimus with a partial remission rate of 15% (95% CI = 0% to 44%; τ2 = 0.0174; I2 = 21%; overall p = 0.26, moderate certainty).

### Adverse effects

A total of 23 studies [27,31–34,36–48,51,52] reported outcomes on adverse events experienced according to different immunosuppressive agents received. A total of 19 events were reported from 34 patients receiving cyclophosphamide in three studies [33,48,51], including infections (5), hepatotoxicity (4), alopecia (2), acute kidney disease (1), neuropathy (1), gastrointestinal symptoms (3), leukopenia (2) and rashes (1). 73 events were reported from three studies [32,33,51] comprising of 29 patients receiving cyclosporine, including hypertension (12), nephrotoxicity (9), interstitial fibrosis (7), vascular lesions (3), gingival hypertrophy (8), tremors (9), hirsutism and hypertrichosis (5), acute kidney disease (5), gout (1), fatigue (1) and hyperglycemia (1). 48 patients receiving mycophenolate mofetil in three studies [33,50,51] experienced a total of 13 adverse events, including gastrointestinal symptoms (11), acute kidney disease (1) and major gastrointestinal bleed (1). 359 patients receiving rituximab reported in 15 studies [27,31,33,34,36–40,42–44,46,47,52] experienced a total of 92 events, including mild infusion reactions such as rashes, throat irritation, chills, itch (54), infections (13), leukopenia (4), hypotension (4), alopecia (1) and ENT symptoms (20). Four studies [33,45,48,51] reporting 31 patients receiving tacrolimus experienced 19 adverse events, including infections (5), hepatotoxicity (1), gastrointestinal symptoms (5), new-onset hypertension (1), reversible nephrotoxicity (2) and diabetes mellitus (2).

## Discussion

Among the five immunosuppressive agents analyzed in this meta-analysis, the pooled evidence suggests that only rituximab has a statistically significant effect in achieving complete remission among patients with SDNS/FRNS. The dosage of rituximab used in the included studies varied, with the majority offering 375 mg/m2 of rituximab for patients, with a cumulative dosage of 500mg to 2g in some studies.

It is also worth noting that there is an I2 > 50% in the pooled results from rituximab and cyclosporine, which suggests that they both have a high heterogeneity. However, as this is a proportional meta-analysis, where little variance is observed even in studies with small sample size, high I^2^ in the context of proportional meta-analysis does not necessarily mean that data is inconsistent, which requires conservative interpretation of test results [55]. Nevertheless, we attribute the cause of heterogeneity to the various subtypes of population, including different tapering steroid dosages and severity of nephrotic syndrome pre-treatment that is difficult to group or uniformize, posing a challenge in this study as individual outcomes for patients are not widely available. The small number of patients within each study further complicates this, while the absence of detailed individual baseline characteristic data for each patient in most studies further complicates the process of conducting subgroup analyses in determining the exact attributing factor. A limited number of studies in the cyclosporine group also led to difficulties in conducting subgroup analysis as investigations of heterogeneity when there are very few studies are of questionable value [56] due to the lack of statistical power [57].

Comparing the adverse effects of the five treatment options, rituximab has the lowest adverse event rate of 0.26, followed by mycophenolate mofetil at 0.27, cyclosporine at 0.39, cyclophosphamide at 0.55 and tacrolimus at 0.61. While the reliability of adverse effect figures may be affected by varying reports from different clinicians, especially in the absence of agreement of what effects to look for between different retrospective studies affecting the accuracy of result [58], the severity of the commonest adverse effects reported among patients receiving each immunosuppressive agent provides an overview of its safety profile. The main adverse effects of rituximab are milder infusion reactions such as rashes, throat irritation, chills, itch whereas both cyclophosphamide and tacrolimus mainly cause infections, cyclosporine mainly causes hypertension and mycophenolate mofetil mainly causes gastrointestinal symptoms.

This side effect profile of rituximab is consistent with a meta-analysis on adult patients by Xue et al., 2020 with steroid-dependent or frequent-relapsing focal segmental glomerulosclerosis or minimal change disease, which found rituximab to be a relatively safe and effective alternative to replace prednisone or calcineurin inhibitors [59]. In Xue’s systematic review and meta-analysis, rituximab was the sole immunosuppressant investigated which had a pooled complete remission rate of 84.2% but high heterogeneity of I^2^ = 83.4%. Compared with this current review, our included studies have a completed remission rate of 89% and heterogeneity of I^2^ = 62.0%. While the differences in percentages may not provide significant evidence, it is worth noting that heterogeneity is present in both reviews, attributable to limited sample size, differing study design and short follow up duration.

Unlike the finding of our study, the children population with steroid dependent or frequently relapsing nephrotic syndrome from a network meta-analysis concluded that cyclophosphamide may be preferred initially while chlorambucil, and rituximab may be acceptable medications for patients with FRNS/SDNS with no significant differences in acceptability found [60]. Moving towards the adult population, to date, there is no network meta-analysis conducted to assess efficacy between different immunosuppressive options due to the lack of randomized controlled trials which necessitates the need for including non-randomized controlled trials requiring adequate control of biases such as confounding to not threaten the validity of the entire meta-analysis [61]. Previous reviews on the adult population focusing on single etiological cause encountered similar challenges, of which one found the possible effectiveness of enteric-coated mycophenolate sodium and calcineurin inhibitors in achieving remission for adults with MCD nephrotic syndrome [62] while another study who only enrolled a small number of participants with FSGS [63] found that cyclosporine may reduce proteinuria among some patients.

Individual multi-arm studies included in our analysis reported varying results when comparing different immunosuppressive agents. Collectively, the findings from this meta-analysis are consistent with one study comparing four immunosuppressive agents [33] which found rituximab may be less likely to require a change of therapy and more likely to come off immunosuppressive drugs as compared to mycophenolate mofetil, calcineurin inhibitors and cyclophosphamide.

Among the four immunosuppressants with non-significant complete remission rates, one study [26] found mycophenolate mofetil to be superior to tacrolimus in maintaining remission while another [48] found tacrolimus accompanied by a tapering dose of prednisolone appears to yield quicker remission than treatment with cyclophosphamide together with prednisone.

With the limitations discussed above, more randomized controlled trials comparing different immunosuppressive agents using standardized dosages within a single study should be carried out. Further studies on adverse effects secondary to the immunosuppressive agents and not disease progression is also necessary to identify the safety profile of immunosuppressive agents used. Only then can the treatment risk be appropriately weighed against the likely benefits according to individual patient factors and drug exposures [20], including comorbid conditions, age, immunological complications and risk of malignancy.

## Conclusion

Among the commonly used immunosuppressive agents, only rituximab has a statistically significant effect in achieving complete remission among patients with SDNS/FRNS and has a relatively good safety profile, but this is limited by low quality of evidence with high degree of heterogeneity causing a lack of statistical power.

## Supporting information

S1 Fig PRISMA 2020 checklist. (PDF)

S2 Fig Funnel plots. (PDF)

S1 Table Search strategies. (PDF)

S2 Table Table of included studies. (PDF)
