# Supplementary material for: Immunosuppression for adult steroid-dependent or frequently relapsing nephrotic syndrome: A systematic review and meta-analysis
Source: PLoS One. 2024 Jul 31;19(7):e0307981. doi: 10.1371/journal.pone.0307981 (PMC11290670; doi:10.1371/journal.pone.0307981)

Supplementary Figure 2 - Funnel Plot

Complete Remission - Cyclophosphamide

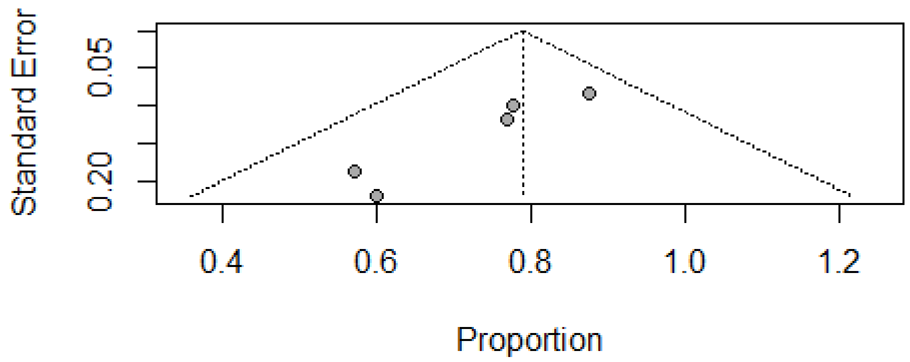

Complete Remission – Cyclosporine

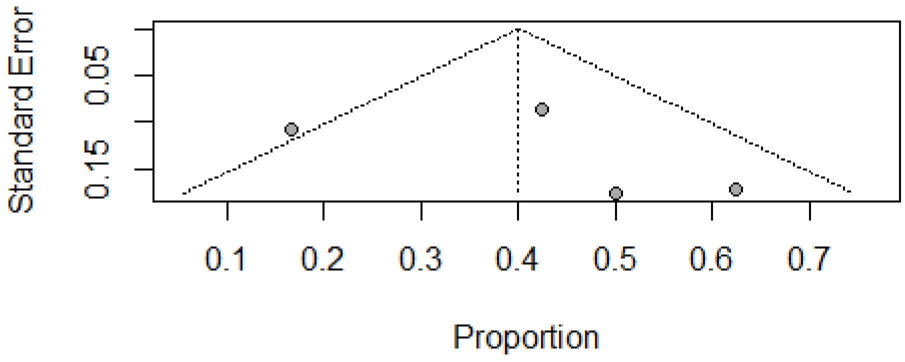

Complete Remission – Mycophenolate Mofetil

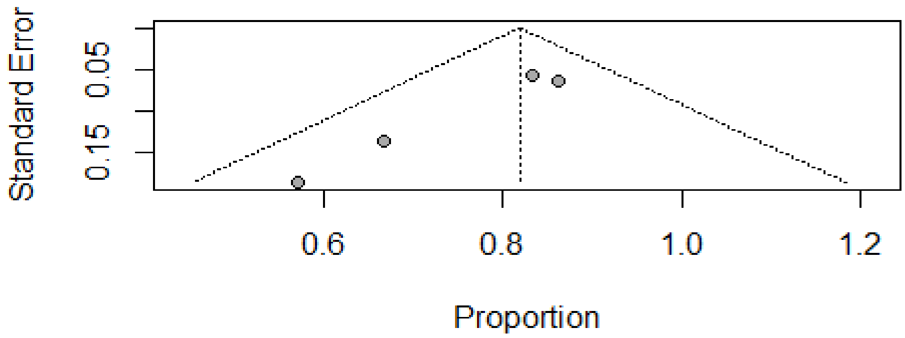

Complete Remission – Rituximab

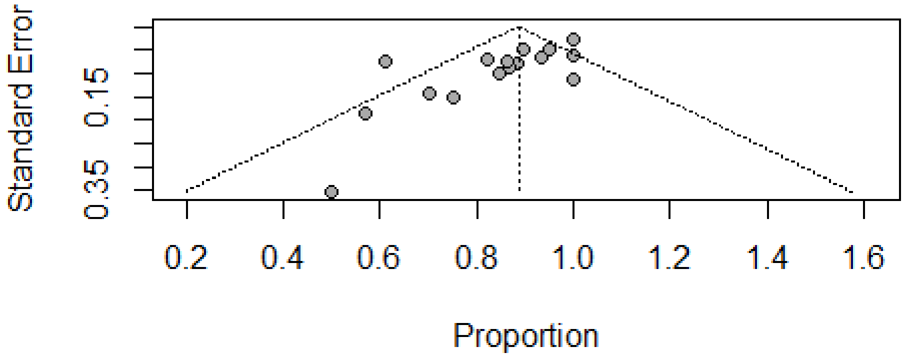

Supplementary Figure 2 - Funnel Plot

Complete Remission – Tacrolimus

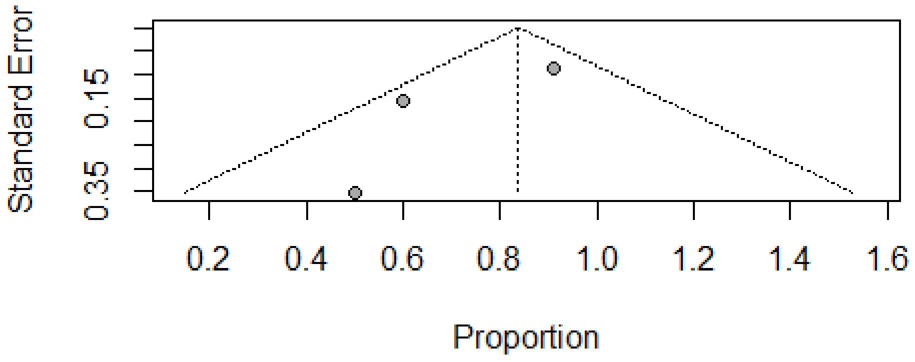

Partial Remission - Cyclophosphamide

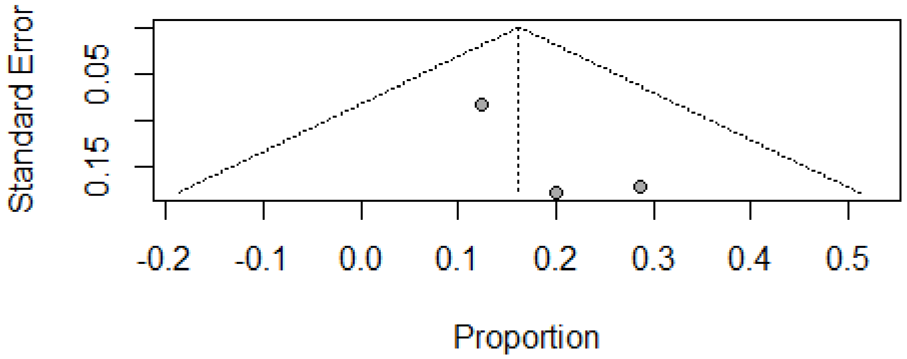

Partial Remission – Cyclosporine

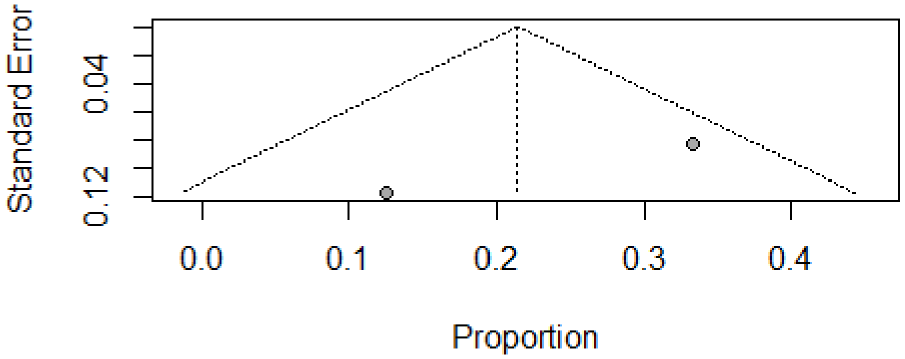

Supplementary Figure 2 - Funnel Plot

Partial Remission – Mycophenolate Mofetil

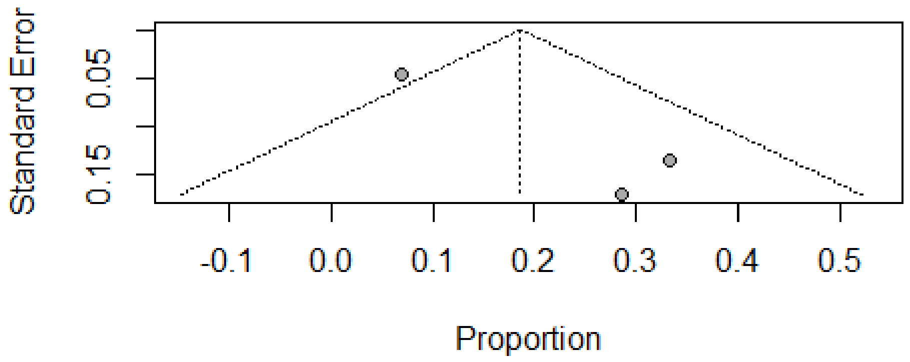

Partial Remission - Rituximab

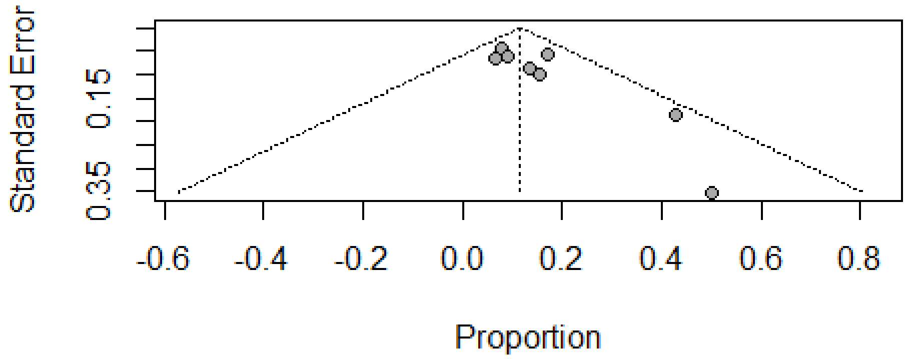

Partial Remission - Tacrolimus

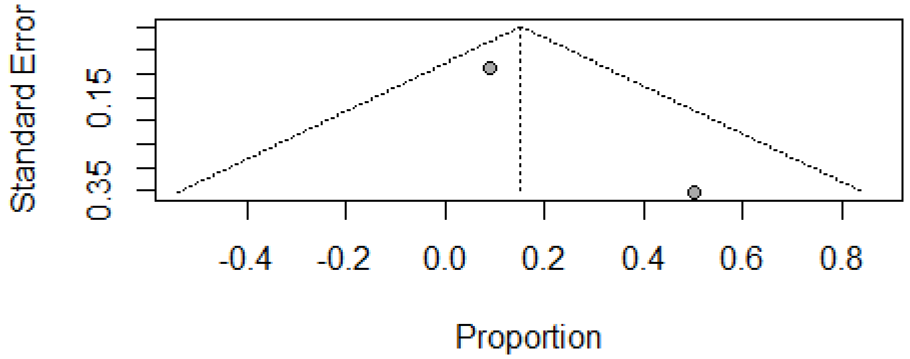

Supplement: S2 Fig — (PDF) [file pone.0307981.s002.pdf]
