# Supplementary material for: Immunosuppression for adult steroid-dependent or frequently relapsing nephrotic syndrome: A systematic review and meta-analysis
Source: PLoS One. 2024 Jul 31;19(7):e0307981. doi: 10.1371/journal.pone.0307981 (PMC11290670; doi:10.1371/journal.pone.0307981)
Supplement: S1 Table — (PDF) [file pone.0307981.s003.pdf]

Supplementary Table 1 - Search Strategies

Database	Search Terms	Results	Last Searched
Embase	1 Nephrotic Syndrome.mp. or exp nephrotic syndrome/<br>2 Lipoid nephrosis.mp. or exp lipoid nephrosis/<br>3 exp minimal change glomerulonephritis/ or exp lipoid nephrosis/ or Minimal change disease.mp. or exp nephrotic syndrome/<br>4 Focal segmental glomerulosclerosis.mp. or exp focal glomerulosclerosis/<br>5 Focal sclerosing glomerulonephritis.mp. or exp focal glomerulosclerosis/<br>6 1 or 2 or 3 or 4 or 5<br>7 Immunosuppression Therapy.mp. or exp immunosuppressive treatment/<br>8 Rituximab.mp. or exp rituximab/<br>9 Mycophenolate.mp. or exp mycophenolic acid/<br>10 inosine monophosphate dehydrogenase.mp. or exp inosinate dehydrogenase/<br>11 Cellcept.mp. or exp mycophenolate mofetil/<br>12 Tacrolimus.mp. or exp tacrolimus/<br>13 Advagraf.mp. or exp tacrolimus/<br>14 Prograf.mp. or exp tacrolimus/<br>15 Envarsus.mp. or exp tacrolimus/<br>16 Cyclosporine.mp. or exp cyclosporin/ or exp cyclosporine/ or exp cyclosporin A/<br>17 Sandimmun.mp. or exp cyclosporine/<br>18 Tacrolimus.mp. or exp tacrolimus/<br>19 Cyclophosphamide.mp or exp cyclophosphamide/<br>20 Endoxan.mp or exp cyclophosphamide/<br>21 Cytoxan.mp or exp cyclophosphamide/<br>22 Neosar.mp or exp cyclophosphamide/<br>23 Procytox.mp or exp cyclophosphamide/<br>24 Sendoxan.mp or exp cyclophosphamide/<br>25 Clafen.mp or exp cyclophosphamide/<br>26 Immunosuppression Therapy.mp. or exp immunosuppressive treatment/<br>27 7 or 8 or 9 or 10 or 11 or 12 or 13 or 14 or 15 or 16 or 17 or 18 or 19 or 20 or 21 or 22 or 23 or 24 or 25 or 26<br>28 exp prednisolone/ or exp steroid/ or exp prednisone/ or steroid-dependent.mp. or exp corticosteroid/<br>29 steroid dependent.mp.<br>30 steroid-sensitive.mp.<br>31 steroid sensitive.mp.<br>32 frequently relapsing.mp<br>33 frequently-relapsing.mp<br>34 28 or 29 or 30 or 31 or 32 or 33<br>35 6 and 27 and 34<br>36 limit 35 to human	3236	01/03/2024

Supplementary Table 1 - Search Strategies

	37 limit 36 to "remove medline records"		
PubMed	((((Nephrotic syndrome[MeSH Terms] OR Nephrosis, Lipoid[MeSH Terms] OR Glomerulosclerosis, Focal Segmental[MeSH Terms])) OR ("Nephrotic Syndrome"[Title/Abstract] OR "Lipoid nephrosis"[Title/Abstract] OR "Minimal change disease"[Title/Abstract] OR "Minimal Change"[Title/Abstract] OR "Minimal change glomerulonephritis"[Title/Abstract] OR "Minimal change nephropat*"[Title/Abstract] OR "Focal segmental glomerulosclerosis"[Title/Abstract] OR "Focal sclerosing glomerulonephritis"[Title/Abstract] OR "FSGS"[Title/Abstract])) AND (("Immunosuppression Therapy"[Title/Abstract] OR Immunosuppressi*[Title/Abstract] OR Immunosuppressan*[Title/Abstract] OR Immunotherap*[Title/Abstract] OR "Immune therap*"[Title/Abstract] OR Rituximab[Title/Abstract] OR MabThera[Title/Abstract] OR Rituxan[Title/Abstract] OR CD20[Title/Abstract] OR "IDEC-C2B8"[Title/Abstract] OR GP2013[Title/Abstract] OR Mycophenolate[Title/Abstract] OR "mycophenolic acid"[Title/Abstract] OR "inosine monophosphate dehydrogenase"[Title/Abstract] OR Cellcept[Title/Abstract] OR Tacrolimus[Title/Abstract] OR Advagraf[Title/Abstract] OR Prograf[Title/Abstract] OR FK506[Title/Abstract] OR "LCP-Tacro"[Title/Abstract] OR LCPT[Title/Abstract] OR Envarsus[Title/Abstract] OR Cyclosporine[Title/Abstract] OR Ciclosporin[Title/Abstract] OR "Cyclosporine-Neoral"[Title/Abstract] OR "Cya-NOF"[Title/Abstract] OR "Cyclosporine A"[Title/Abstract] OR Neoral[Title/Abstract] OR "OL-27-400"[Title/Abstract] OR Sandimmun[Title/Abstract] OR "Sandimmun Neoral"[Title/Abstract] OR “cyclophosphamide”[Title/Abstract] OR “endoxan”[Title/Abstract] OR “cytoxan”[Title/Abstract] OR “Neosar”[Title/Abstract] OR “Procytox”[Title/Abstract] OR “Sendoxan”[Title/Abstract] OR “Clafen”[Title/Abstract]) OR (Immunosuppression Therapy[MeSH Terms] OR Rituximab[MeSH Terms] OR Mycophenolic Acid[MeSH Terms] OR Tacrolimus[MeSH Terms] OR Cyclosporine[MeSH Terms] OR Cyclosporins[MeSH Terms] OR Cyclophosphamide[MeSH Terms]))) AND ("steroid-dependent"[Title/Abstract] OR "steroids-dependent"[Title/Abstract] OR "steroid dependent"[Title/Abstract] OR "steroids dependent"[Title/Abstract] OR "steroid-sensitive"[Title/Abstract] OR "steroid	793	03/03/2024

Supplementary Table 1 - Search Strategies

	sensitive"[Title/Abstract] OR “frequently-relapsing”[Title/Abstract] OR “frequently relapsing”[Title/Abstract])		
Web Of Science	"Nephrotic Syndrome" OR "Lipoid nephrosis" OR "Minimal change disease" OR "Minimal Change" OR "Minimal change glomerulonephritis" OR "Minimal change nephropat*" OR "Focal segmental glomerulosclerosis" OR "Focal sclerosing glomerulonephritis" OR "FSGS" (Topic) and "Immunosuppression Therapy" OR Immunosuppressi* OR Immunosuppressan* OR Immunotherap* OR "Immune therap*" OR Rituximab OR “MabThera” OR Rituxan OR CD20 OR "IDEC-C2B8" OR GP2013 OR Mycophenolate OR "mycophenolic acid" OR "inosine monophosphate dehydrogenase" OR Cellcept OR Tacrolimus OR Advagraf OR Prograf OR FK506 OR "LCP-Tacro" OR "LCPT" OR "Envarsus" OR Cyclosporine OR Ciclosporin OR "Cyclosporine-Neoral" OR "Cya-NOF" OR "Cyclosporine A" OR "Neoral" OR "OL-27-400" OR Sandimmun OR "Sandimmun Neoral" OR "cyclophosphamide" OR "endoxan" OR "cytoxan" OR "Neosar" OR "Procytox" OR "Sendoxan" OR "Clafen" (Topic) and "steroid-dependent" OR "steroids-dependent" OR "steroid dependent" OR "steroids dependent" OR "steroid-sensitive" OR "steroid sensitive" OR "frequently-relapsing" OR "frequently relapsing" (Topic)	839	03/03/2024
Cochrane Central Register of Controlled Trials (CENTRAL)	"Nephrotic Syndrome" OR "Lipoid nephrosis" OR "Minimal change disease" OR "Minimal Change" OR "Minimal change glomerulonephritis" OR "Minimal change nephropat*" OR "Focal segmental glomerulosclerosis" OR "Focal sclerosing glomerulonephritis" OR "FSGS" in Title Abstract Keyword AND "Immunosuppression Therapy" OR Immunosuppressi* OR Immunosuppressan* OR Immunotherap* OR "Immune therap*" OR Rituximab OR “MabThera” OR Rituxan OR CD20 OR "IDEC-C2B8" OR GP2013 OR Mycophenolate OR "mycophenolic acid" OR "inosine monophosphate dehydrogenase" OR Cellcept OR Tacrolimus OR Advagraf OR Prograf OR FK506 OR "LCP-Tacro" OR "LCPT" OR "Envarsus" OR Cyclosporine OR Ciclosporin OR "Cyclosporine-Neoral" OR "Cya-NOF" OR "Cyclosporine A" OR "Neoral" OR "OL-27-400" OR Sandimmun OR "Sandimmun Neoral" OR "cyclophosphamide" OR "endoxan" OR "cytoxan" OR "Neosar" OR "Procytox" OR "Sendoxan" OR "Clafen" in Title Abstract Keyword AND "steroid-dependent" OR "steroids-dependent" OR "steroid dependent" OR "steroids dependent" OR "steroid-sensitive" OR "steroid sensitive" OR "frequently-relapsing" OR "frequently	240	03/03/2024

Supplementary Table 1 - Search Strategies

	relapsing" in Title Abstract Keyword - (Word variations have been searched)		
