# Supplementary material for: Immunosuppression for adult steroid-dependent or frequently relapsing nephrotic syndrome: A systematic review and meta-analysis
Source: PLoS One. 2024 Jul 31;19(7):e0307981. doi: 10.1371/journal.pone.0307981 (PMC11290670; doi:10.1371/journal.pone.0307981)
Supplement: S2 Table — (PDF) [file pone.0307981.s004.pdf]

Study	Study Design	Country	Funding	Inclusion Criteria	Exclusion Criteria	Sample Size	Mean Age (Years)	Mean Follow-Up Duration (Months)	Serum creatinine level (Baseline)	Serum albumin (g/L)	Previous Therapies		Dosage	Partial remission (Fraction)	Complete/ Full remission (Fraction)	Duration for Remission (Days)	Rate of Relapse (%)	Adverse events (Overall Study)	Nephrotic Syndrome Definition
Aarthi et al., 2019	Retrospective Study	India	-	Adults with SDNS from January 2018 to December 2018	Patients with SRNS	42	16.5 ± 2.3	6.5 ± 1.6				Mycophenolate mofetil			35/42		9	29 subjects including abscess, diarrhea, urinary tract infection, peritonitis, pulmonary tuberculosis, and herpes zoster	
						10						Tacrolimus			6/10		30		
						18						Cyclophosphamide			14/18		16		
Bruchfeld et al., 2014	Prospective Study	Sweden	-	Patients 27 - 70 years with renal biopsy proven MCN		15	39.8	37.5		26.3	Glucocorticoids (14/15), CS (1/15), Cyclophosphomide (4/15), Cyclosporine (2/15), Levamisole (2/15), Tacrolimus (1/15), MMF (3/15)	Rituximab	Initial protocol of 500 mg 2 weeks apart for the first nine patients, one was given 375 mg/m2 at three time-points, with a total dose of 1500 mg. Three patients were given a rituximab dose of 375 mg/m2 once weekly for 4 weeks, two patients 1000 mg 2 weeks apart. Total rituximab exposure varied between 1000 and 2800 mg.	2/15	13/15	43	47	Transient hypotension (2), Itchy and red eyes (1)	-
Cortazar et al., 2018	Retrospective Study	USA	Genentech	Patients >18 years with biopsy proven MCD or primary FSGS and a urinary protein:creatinine ratio (UPCR) of >3.5 g/g at initiation of treatment.	Patients with identifiable secondary cause of FSGS	13	52.8	45.7	1.28 mg/dL	25.1	Prednisone (13/13), Cyclophosphomide (2/13), Azathioprine (1/13), Tacrolimus (3/13), MMF (6/13), Cyclosporine A (5/13), Abatecept (1/13)	Rituximab	Two 1000mg IV doses separated by 2–4 weeks. Thereafter, one 1000mg IV dose every 4 months	2/13	11/13		0		UPCR >3.5 g/g, serum albumin (Alb) <3.5 mg/dL and edema
DaSilva et al., 2017	Retrospective Study	Spain	None	Adults with SDNS and FRNS with biopsy-proven MCD, MesGN or FSGS requiring multiple immunosuppressants to maintain remission	Patients with systemic diseases, including HIV, hepatitis B and C; secondary FSGS, steroid resistant INS or kidney transplant	28	37 ± 15	31 ± 26	74 ± 32 µmol/L	19.7 ± 4.8 g/L	Cyclosporine (20/28), Tacrolimus (18/28), Mycophenolate mofetil (22/28), Chlorambucil (3/28), Cyclophosphamide (7/28)	Rituximab	One (10), two (9), three (5) or four (4) infusions of rituximab 375 mg/m2.		23/28				KDIGO guideline
Girimaji et al., 2021	Retrospective Study	India	-	Patients with biopsy-proven cFSGS	Patients with HIV, Hepatitis B and Hepatitis C infections	2	41	15	1.25 mg/dL	38.5	Tacrolimus (1/2), Cyclosporine (1/2)	Rituximab	Initial dose of 375 mg/m2; patients having CD-19 levels >5/µL or >1% at 1 month received additional low-dose 100 mg of rituximab	1/2	1/2			Mild infusion reactions (3), Upper respiratory Tract Infection (2)	-
Gorsane et al., 2016	Retrospective Study	Tunisia	-	Patients with histologically proven FSGS	-	8	21 ± 8.9	6.8 ± 3.7 (Years)	95.25 µmol/L	16.93	Corticosteroids	Cyclosporine A	Starting 4 mg/kg per day, most often in two divided doses to achieve a target level then dosage adjusted to maintain levels of 80–150 ng/mL. After remission, adjustments only when trough levels were >150 ng/mL. Patients with episodic relapses were maintained at higher CsA levels (100–150 ng/mL)	1/8	4/8			Hypertension (11), nephrotoxicity (9), interstitial fibrosis (7), vascular lesions (3), gingival hypertrophy (8), tremors (8), hirsutism and hypertrichosis (5)	-
Heybeli et al., 2021	Retrospective Study	USA	-	Patients with MCD based on a kidney biopsy examination	MCD related to secondary disorders including infection or cancer, MCD without frequent relapse or steroid dependence, steroid resistant MCD, diagnosis of FSGS in a subsequent kidney biopsy, histological diagnosis of other disorders concurrently with MCD	13	46	81	0.6–4.2 mg/dL	9 - 31	Corticosteroids	Rituximab	1 g of RTX 15 days apart (totally 2 g)	2/13	11/13			Post-infusion side effects (3), rash (1), back pain (1), nausea (1)	-
						12						Mycophenolate mofetil		4/12	8/12			Gastrointestinal intolerance (3), acute kidney disease (1)	
						16						Cyclophosphamide		2/16	14/16			Leukopenia (2), allopecia (2), acute kidney disease (1), neuropathy (1), worsening gastroesophageal reflux (1), rash (1)	
Katsuno et al., 2019	Retrospective Study	Japan	Chugai Pharmaceutical Co., Ltd., Daiichi Sankyo Co., Ltd., Eisai Co., Ltd., Kyowa Hakko Kirin Co., Ltd., Mitsubishi Tanabe Pharma Clinical and Experimental Nephrology1 3Co., Novartis Pharma K.K., Otsuka Pharmaceutical Co., Ltd., Pfizer Japan Inc., Takeda Pharmaceutical Co., Ltd., Teijin Pharma Limited., and Torii Pharmaceutical Co., Ltd. The Department of Nephrology, Nagoya University Graduate School of Medicine received research promotion grants from Astellas Pharma Inc., Bristol-Myers Squibb., Chugai Pharmaceutical Co., Ltd., Daiichi Sankyo Co., Kissei Phar-maceutical Co., Ltd., Kowa Pharmaceutical Co., Ltd., Kyowa Hakko Kirin Co., Ltd., Mitsubishi Tanabe Pharma Co., Mochida Pharmaceu-tical Co., Ltd., MSD K.K., Nippon Boehringer Ingelheim Co., Ltd., Novartis Pharma K.K., Otsuka Pharmaceutical Co., Ltd., Pfizer Japan Inc., Takeda Pharmaceutical Co., Ltd., Teijin Pharma Limited., Torii Pharmaceutical Co., Ltd., and Sumitomo Dainippon Pharma Co., Ltd	Adult-onset SDNS patients who were administered rituximab	-	8	30 – 49.5	11.6 – 20.0	0.61–0.99 mg/dL	33 – 40	Prednisolone (8), cyclosporine (8), tacrolimus (1), mizoribine (3), MMF (2), cyclophosphomide (1)	Rituximab	Single dose 500 mg rituximab (6), 7 infusions totaling 3100 mg of rituximab (1) and 3 infusions totaling 1500 mg of rituximab (1)		6/8			Infusion reaction - hypotension (1)	-
Keskar et al., 2013	Retrospective Study	India	-	Patients > 12 years with MCD and had at least one outpatient department visit in the preceding 6 months	Patients withsecondary glomerular disorders	7	30.46 ± 13.43	4 - 97.5	1.16 mg/dL	20 ± 7.1	Corticosteroids	Cyclophosphamide	4480–10752 mg over mean duration of 10 weeks	2/7	4/7		44.26	AKI (14), Varicella zoster, spontaneous bacterial peritonitis and pulmonary tuberculosis	-
Kidd & Bean, 2018	Retrospective Study	USA	-	Steroid-dependent MCD patients		7	23 - 62	2 - 37				Rituximab	1000 mg IV spaced 2 weeks apart	3/7	4/7			Alopecia (1)	UP/C > 3.5, hypoalbuminemia and edema
Brown et al., 2017	Retrospective Study	USA	Howard Holderness Distinguished Medical Scholars Program research fellowship grant, NIDDK, The Broad Institute	Patients 18 years or older, administered an initial dose of rituximab between 2009 and 2014 for treatment of corticosteroid-dependent minimal-change glomerulopathy	Prior rituximab therapy or minimal-change disease that was deemed to be secondary to another process	5	55.4	39.5	0.98 (0.81-1.24)	2.5 (1.9-4)	Corticosteroids (5), Cyclosporine (5), Tacrolimus (1), Cyclophosphomide (2), MMF (3)	Rituximab	2 doses of 1,000 mg spaced 2 to 3 weeks apart (2 dose regimen) or 4 doses of 375 mg/m2 spaced 1 week apart (4 dose regimen)		5/5	30 - 90		Upper respiratory infection (1), Disorientation and confusion 2 weeks after infusion (1)	-
Dekkers et al., 2015	Retrospective Study	Netherlands		Patient with SD MCN	-	10	26.4 ± 13.21	43 ± 23.5			Prednisone (7), cyclophosphamide (4), cyclosporine (6), mycophenolate mofetil (1), levamisole (2)	Rituximab	Two doses of rituximab (375 mg/m2)		7/10			None	-
Guitard et al., 2014	Retrospective Study	France	-	Adult patients who presented with biopsy proven MCNS and received RTX after 15 years	Biopsy performed within first 5 years showed signs of FSGS, patients who received RTX to treat a disease other than MCNS, patients with concomitant thymoma or patients with SRNS	41	26 (15-83)	92 ± 26	1.04 ± 0.29	3.2 ± 0.9	Steroids (37), CNI (21), MMF (11)	Rituximab	1 g on Days 1 and 15 (21), four weekly infusions of 375 mg/m² (12), 1 g once (1), two weekly infusions of 375 mg/m² (5), three weekly infusions of 375mg/m² (2)	7/41	25/41			Non significant	albuminemia <30 g/L and high-range proteinuria (uPr/Cr >3 g/g)
Miyabe et al., 2016	Prospective Study	Japan	-	Patients with SDNS with biopsy-proven MCD diagnosis	Patients with systemic diseases, hepatitis B and C, HIV and antinuclear antibodies, positive family history and previous history of rituximab treatment	10	28.2 ± 10.4	24	0.7 ± 0.02	3.7 ± 0.08		Rituximab	A single dose [375 mg/m2 BSA (max 500 mg)] of rituximab administered four times at an interval of 6 months		10/10			Itching (10), Pharyngalgia (5), Cutaneous rash (7), Decreased blood pressure (1), Ear fullness (1), Nasal obstruction (1)	urinary protein excretion level of >3.5 g/day, serum albumin level of <3.0 g/dL, edema, and hyperlipidemia
Munyentwali et al., 2013	Retrospective Study	France		Patients with SDNS or FRNS treated with rituximab	Patients with histopathological diagnosis of focal and segmental glomerulosclerosis	17	29.4 (18.5-65)	29.5 (5.1-82.2)	79.1 µmol/L	3.3 (2.2-3.8)	Levamisole (7), cyclophosphamide (4), cyclosporine A (13), mechlorethamine (1), MMF (12), Basiliximab (1), Car (3), chlorambucil (2), pefloxacin (1), tacrolimus (2)	Rituximab	One (1), two (7), three (4) or four (3) weekly rituximab infusions of 375 mg/m2. Two fixed doses of 1000 mg on days 1 and 15 (2)		15/17			None	-

Ren et al., 2017	Prospective Study	China	Shanghai Jiaotong University School of Medicine multi-center clinical research program, National Basic Research Program of China 973, National Basic Research Program of Shanghai, National Key Research and Development Program of China, National Natural Science Foundation of China	Patients with FRNS who had renal biopsy-proven FSGS or MCD, exhibited evidence of long-term treatment with immunosuppressive drugs, GFR higher than 60 ml/min	Patients with heriditary or secondary FRNS	15	25(16-54)	8(3-36)	0.71 (0.48-0.88)+61:61	3.7 (1.4-4.5)		Rituximab	375 mg/m2 intravenously on days 1, 8, 23, and 29	2/15	13/15			None serious	-
Takei et al., 2013	Prospective Study	Japan	-	Patients with SDNS, biopsy-proven MCD	Associated systemic disease; negative serology for hepatitis B and C, HIV and antinuclear antibodies; no positive family history	25	30±12	12	0.7±0.2	3.4±0.8		Rituximab	Intravenous injection at a single dose of 375 mg/m2 body surface area (BSA) (maximum, 500 mg) and another of same dose at 6 months		25/25		24	None severe	proteinuria ≥3.5 g/day, serum albumin <3.0 g/dL, oedema and hyperlipidaemia
Li et al., 2021	Retrospective Study	China		Steroid-dependent including frequent relapse; all patient had been received steroids in the past		20	21 ± 9	15.5 ± 7.0	76±38μmol/L	33±10g/L	Mycophenolate mofetil (14/20), Steroids (20/20)	Rituximab	Single-dose rituximab (1g)		19/20		25	Mild infusion reaction - skin itching, rash (3), pneumocystis pneumonia (1), skin infection caused by actinomycetes (1)	-
Li et al., 2008	Prospective Study	China		Patients with biopsy-proven minimal change disease, nephrotic syndrome, steroid dependence, Scr < 133μmol/L	Systemic diseases, active infection, abnormal glucose tolerance test, liver function test abnormalities, active peptic ulcer disease and previous therapy with CYC, MMF and CYA (other than corticosteroids)	13	33.8 ± 12.1	22.2 ± 9.9	82.1 ± 24.8	18.9 ± 5.1 g/L	Cyclophosphamide, cyclosporin, mycophenolate mofetil	Cyclophosphamide	750 mg/m2 body surface once every 4 weeks		10/13	59.9 ± 28.3	40	Infection (4), Hepatotoxicity (4), Gastrointestinal symptoms (2)	nephrotic-range proteinuria >3.5 g/24 h with serum albumin < 30 g/l), steroid dependence and serum creatinine (Scr) < 133 μmol/l.
						11	25.0 ± 10.5	23.7 ± 10.7	86.0 ± 29.7	16.7 ± 7.1 g/L		Tacrolimus	0.05 mg/kg/day, divided into two doses over 12-h intervals		10/11	31.5 ± 25.8	50	Infection (3), Hepatotoxicity (1), Gastrointestinal symptoms (2), New-onset hypertension (1)	
Ramachandran et al., 2020	Prospective Study	India	Indian Council of Medical Research and PGIMER-intramural fund	Adults between 16 and 60 years of age with SD or FR or SR NS (but CNI dependent) due to podocytopathy (MCD/FSGS) were included.	Patients with membranous nephropathy, diabetes mellitus, seropositivity for hepatitis B/C and HIV, active infection, pregnancy and known malignancy were excluded.	39	30 (18,45)	36	0.80 (0.80, 0.90) mg/dl	3.80 (3.60, 4.04) g/dL		Rituximab	administrations of 1 g of rituximab, 2 weeks apart.	3/39	35/39			Minor infusion reaction (20), upper respiratory tract infection (7)	proteinuria ≥3.5 g/day along with hypoalbuminemia and oedema
Ramachandran et al., 2019	Prospective Study	India	-	Adult (16–60 years) patients with CNI-dependent NS due to podocytopathy (MCD/FSGS) (diagnosed on light microscopy, immunofluorescence and electron microscopy) were included	Patients with an estimated glomerular filtration rate (eGFR) of <60 mL/min per 1.73 m2, diabetes mellitus (DM), seropositivity for hepatitis B/C and HIV, active infection, pregnancy, known malignancy and prior therapy with either CYC, MMF, azathioprine or rituximab were excluded	15	24	12				Rituximab	375 mg/m2 at 0 m and CD19 targeted fixed dose (100 mg)	1/15	14/15				proteinuria ≥3.5 g/day or ≥ 1.5 g/day along with serum albumin <2.5 g/dL, edema, and hyperlipidemia
Ramachandran et al., 2015	Prospective Study	India	-	Adult-onset SD-MCD cases, which relapsed despite CYC and were treated with TAC monotherapy		11					Cyclophosphamide	Tacrolimus	C0-5-10 ng/ml	1/11	10/11		36%	Reversible nephrotoxicity (2), TAC related diarrhoea (3), infections (2), diabetes mellitus (2)	-
Sandoval et al., 2017	Retrospective Study	Spain	-	Adult patients having a nephrotic syndrome relapse in the setting of SD/FR-INS.		29	40	32.8	70–90 μmol/L		Received previous standard first-line therapy with prednisone (PDN) at 1 mg/kg/day over a minimum period of 1 month.	Mycophenolate mofetil	Starting doses of MF were 2000 mg/day for mofetil MF (1500 mg/day in one patient) or 1440 mg/day for sodium MF.	2/29	25/29		45%	Minor digestive disorders - abdominal pain and/or soft faeces (4)	KDIGO
Waldman et al., 2007	Retrospective Study	USA	Glomerular Centre at Columbia University and Zo's Fund for Life	Adults who had biopsy-proven MCD and were followed at Columbia University Medical Center from 1990 to 2005	Age < 18 years at the time of biopsy or had a biopsy or began treatment before 1990.	5	45.1±1.6 year	132.1 week	1.39 ± 0.13 mg/dl	2.21 ± 0.08 g/dl		Cyclophosphamide	123.6 mg/d	1/5	3/5	5 - 12 weeks		Herpes zoster with transverse myelitis (1)	-
						8						Cyclosporine	220 mg /d (concentration 150 to 200 ng/ml) given in divided dose	1/8	5/8	2 - 9 weeks	41%	None	
						2						Tacrolimus	2 to 4 mg twice daily; trough concentrations 5 to 10 ng/ml	1/2	1/2			None	
						7					Cyclophosphamide and/or cyclosporine (10)	Mycophenolate mofetil	2 g/d	2/7	4/7	20 ± 2.7 weeks		Nausea (2), diarrhea (2), major gastrointestinal bleed (1)	
Zhao et al., 2022	Retrospective Study	China	Natural Science Foundation of China	Patients with steroid dependent nephrotic syndrome of biopsy-proven MCD or primary FSGS		12						Cyclosporine	100 mg/day - 8/12 ; 150 mg/day - 3/12; 200 mg/day - 1/12		2/12	10.4 months	41%	Skin rash and diarrhea (1) ; Skin rashes (3); elevated blood pressure of <160/100 mmHg (2)	-
Iwabuchi et al., 2014	Prospective Study	Japan		Patients with steroid dependent nephrotic syndrome	Patients with no known associated systemic disease, including negative serology for hepatitis B and C, HIV and antinuclear antibodies, and no positive family history; no previous history of rituximab treatment.	25	30.1 ± 11.9	36	0.7 ± 0.2 mg/dL	3.6 ± 0.8 g/dL	CyA (20), Mizoribine (6), MMF (1), TAC (1), Steroid (28)	Rituximab	375 mg/m2 BSA once a week for 4 weeks		25/25	36 to 54 months		Exanthema (1), Leukopenia (1), Cough and hiccups (13)	
Jafry et al., 2023	Retrospective Study	Pakistan	None	Patients ≥16 years presenting to the nephrology outpatient department of Sindh Institute of Urology and Transplantation (SIUT), Karachi, Pakistan between January 1995 and June 2018 with a minimum of 1 year of follow-up, only SDNS patients are included for this meta-analysis	Patients with secondary FSGS	33	25.9 ± 9.6	197.0 ± 105.9 weeks	1.0 ± 0.6 mg/dL	2.5 ± 2.3 g/dL	Corticosteroids	Cyclosporine	4 mg/kg/day	11/33	14/33			Acne (17), weight gain (14), gastric discomfort (9), hypertension (6), diabetes (4), proximal myopathy (3), cataract (1) psychosis (1)	
Zhang et al., 2023	Retrospective Study	China	Scientific Research Management Project of Health Commission of Gansu Province	Patients aged > 18 years with biopsy-confirmed MCD		22	20.2–60.3	10 – 24	73.2 ± 24.4	28.1 ± 9.9 g/L	Corticosteroid (22), Cyclosporine (1), Tacrolimus (1), Cyclophosphamide (3), Myocophenalate Mofetil (1)	Rituximab	4 weekly infusions of 200 mg	2/22	19/22			Allergic reaction during first infusion (fever, chills, bronchospasm, skin rash, hypotension)	Urinary protein excretion of > 3.5 g/day, serum albumin of <3.0 g/dL, edema, and hyperlipidemia
